# Supplementary material for: The complete genome of Banana streak GF virus Yunnan isolate infecting Cavendish Musa AAA group in China
Source: PeerJ. 2020 Jan 27;8:e8459. doi: 10.7717/peerj.8459 (PMC6991131; doi:10.7717/peerj.8459)
Supplement: Table S1 [file peerj-08-8459-s001.docx]

**Supplemtary Table S1.** Sequence identities between BSGFV-YN and other badnaviruses

| **Virus** | **Genome size (bp)** | **Nucleotide identity of complete genome (%)** | **Nucleotide acid/amino acid (%)** | | |
| --- | --- | --- | --- | --- | --- |
|  |  |  | ***ORF*Ⅰ/ ORFⅠ** | ***ORFⅡ* / ORFⅡ** | ***ORFⅢ*** **/ ORFⅢ** |
| BSGFV-YN | 7325 | 100.00 | 100/100 | 100/100 | 100/100 |
| BSGFV | 7263 | 98.14 | 99.62/99.43 | 98.82/99.11 | 99.02/98.91 |
| BSIMV | 7769 | 54.57 | 51.97/50.84 | 49.37/31.06 | 59.25/56.42 |
| BSMYV | 7652 | 54.47 | 52.26/46.59 | 47.01/33.08 | 57.88/52.17 |
| BSOLV | 7398 | 55.57 | 51.21/46.37 | 36.70/23.70 | 59.13/55.16 |
| BSUIV | 7458 | 50.72 | 51.06/40.64 | 43.64/24.35 | 52.43/42.42 |
| BSULV | 7401 | 51.02 | 50.88/39.04 | 44.29/18.10 | 52.69/42.83 |
| BSUMV | 7532 | 49.10 | 50.26/40.43 | 42.06/21.74 | 52.09//42.75 |
| BSVNV | 7797 | 54.15 | 52.35/50.28 | 42.08/29.85 | 59.92/55.65 |
| BSUAV | 7519 | 57.09 | 60.04/56.18 | 37.44/19.23 | 59.21/55.91 |
| BSV-Acum | 7722 | 54.61 | 51.22/51.41 | 42.29/29.10 | 59.34/55.51 |
| ComYMV | 7489 | 46.07 | 45.17/34.65 | 40.23/18.98 | 44.41/37.38 |
| CaYMV | 7348 | 53.58 | 51.38/42.46 | 40.00/32.84 | 56.68/53.85 |
| PBCOV | 7543 | 51.49 | 48.15/43.33 | 44.03/35.48 | 52.95/48.25 |
| SBMOV | 7568 | 49.16 | 50.09/43.32 | 48.39/27.20 | 51.98/41.96 |
| KTSV | 7591 | 52.92 | 44.91/30.29 | 44.53/28.23 | 55.81/51.28 |
