# Supplementary material for: The complete genome of Banana streak GF virus Yunnan isolate infecting Cavendish Musa AAA group in China
Source: PeerJ. 2020 Jan 27;8:e8459. doi: 10.7717/peerj.8459 (PMC6991131; doi:10.7717/peerj.8459)
Supplement: Table S2 [file peerj-08-8459-s002.docx]

**Supplemtary Table S2.** The amino acid sequence identities of the putative domains between BSGFV-YN ORF**Ⅲ** and other badnaviruses ORF**Ⅲ***s*

| **Virus** | **Amino acid sequence identity of the putative domains (%)** | | | | | | |
| --- | --- | --- | --- | --- | --- | --- | --- |
|  | **MP** | **CP** | **AP** | **RT** | **RNaseH** | **1^st^ CR** | **2^nd^ CR** |
| BSGFV-YN | 100.00 | 100.00 | 100.00 | 100.00 | 100.00 | 100.00 | 100.00 |
| BSGFV | 100.00 | 98.21 | 98.98 | 100.00 | 98.45 | 100.00 | 100.00 |
| BSIMV | 74.51 | 70.71 | 64.29 | 84.29 | 70.54 | 77.78 | 59.26 |
| BSMYV | 85.42 | 61.79 | 49.49 | 77.49 | 65.89 | 83.33 | 53.33 |
| BSOLV | 70.59 | 66.79 | 53.06 | 82.20 | 72.87 | 72.22 | 44.44 |
| BSUIV | 58.33 | 51.79 | 39.39 | 76.96 | 61.24 | 62.50 | 22.22 |
| BSULV | 60.42 | 50.71 | 43.43 | 75.92 | 62.79 | 62.50 | 50.00 |
| BSUMV | 62.50 | 49.64 | 39.39 | 77.49 | 62.79 | 62.50 | 50.00 |
| BSVNV | 74.51 | 71.79 | 66.33 | 84.29 | 69.77 | 77.78 | 51.85 |
| BSUAV | 72.55 | 72.14 | 64.29 | 81.15 | 71.32 | 88.89 | 66.67 |
| BSV-Acum | 72.55 | 71.79 | 66.33 | 83.25 | 69.77 | 77.78 | 39.39 |
| ComYMV | 72.92 | 39.58 | 41.41 | 76.44 | 67.44 | 61.11 | 80.00 |
| CaYMV | 79.17 | 60.71 | 47.47 | 82.20 | 68.22 | 66.67 | 55.56 |
| PBCOV | 79.17 | 60.00 | 44.44 | 79.58 | 71.32 | 61.11 | 60.00 |
| SBMOV | 47.92 | 49.64 | 41.00 | 72.25 | 58.14 | 57.14 | 80.00 |
| KTSV | 71.15 | 68.21 | 64.29 | 82.72 | 69.77 | 72.22 | 75.00 |
